# A model of human lung fibrogenesis for the assessment of anti-fibrotic strategies in idiopathic pulmonary fibrosis

**DOI:** 10.1038/s41598-017-18555-9

**Published:** 2018-01-10

**Authors:** Katy M. Roach, Amanda Sutcliffe, Laura Matthews, Gill Elliott, Chris Newby, Yassine Amrani, Peter Bradding

**Affiliations:** Institute for Lung Health, Respiratory Medicine, Department of Infection, Immunity and Inflammation, University of Leicester, Leicester, UK

## Abstract

Idiopathic pulmonary fibrosis (IPF) is a progressive interstitial lung disease with limited therapeutic options. K_Ca_3.1 ion channels play a critical role in TGFβ1-dependent pro-fibrotic responses in human lung myofibroblasts. We aimed to develop a human lung parenchymal model of fibrogenesis and test the efficacy of the selective K_Ca_3.1 blocker senicapoc. 2 mm^3^ pieces of human lung parenchyma were cultured for 7 days in DMEM ± TGFβ1 (10 ng/ml) and pro-fibrotic pathways examined by RT-PCR, immunohistochemistry and collagen secretion. Following 7 days of culture with TGFβ1, 41 IPF- and fibrosis-associated genes were significantly upregulated. Immunohistochemical staining demonstrated increased expression of ECM proteins and fibroblast-specific protein after TGFβ1-stimulation. Collagen secretion was significantly increased following TGFβ1-stimulation. These pro-fibrotic responses were attenuated by senicapoc, but not by dexamethasone. This 7 day *ex vivo* model of human lung fibrogenesis recapitulates pro-fibrotic events evident in IPF and is sensitive to K_Ca_3.1 channel inhibition. By maintaining the complex cell-cell and cell-matrix interactions of human tissue, and removing cross-species heterogeneity, this model may better predict drug efficacy in clinical trials and accelerate drug development in IPF. K_Ca_3.1 channels are a promising target for the treatment of IPF.

## Introduction

Idiopathic pulmonary fibrosis (IPF) is a common, progressive and invariably fatal lung disease^1–3^. Median survival is 3 years^4^, and there are currently only two licensed treatments, pirfenidone^5^ and nintedanib^6^. The mechanism of action of pirfenidone is poorly understood, while nintedanib is an inhibitor of PDGF/VEGF/FGF receptor tyrosine kinases^6,7^. Both drugs are of limited efficacy^6,7^ and often poorly tolerated. More effective treatments are therefore required urgently.

The causes of IPF remain unresolved, but evidence favours alveolar epithelial cell injury as the initial event which triggers the release of TGFβ1^8^, a central pro-fibrotic growth factor driving lung parenchymal fibrosis^9,10^. Working in concert with additional growth factors including PDGF^11^ and FGF2^12^, TGFβ1 drives the formation of fibroblast foci and the exaggerated deposition of extracellular matrix (ECM). Repeated injury at different sites within the lung generates multifocal areas of pathology at different stages of development^13^.

Animal models make an important contribution to the study of disease mechanisms and therapeutic strategies. However modelling human disease is extremely difficult, and this appears particularly so for IPF^14^. Major discrepancies between drug effects in the bleomycin mouse model of lung fibrosis and human clinical trials for IPF are evident^15^. Until 2006, 246 experimental studies describing beneficial anti-fibrotic compounds in the bleomycin model were reported^16^, but to-date, only 2 drugs have been licenced. There is therefore a need for additional models of human lung fibrogenesis which can help predict drug efficacy in IPF.

*Ex vivo* models of human tissue are already used for pharmacological studies. Precision-cut lung slices and airway organ baths are useful for investigating airway biology^17,18^, while *ex vivo* culture of human prostate tissue has provided clinically relevant insights into prostate carcinogenesis^19^. A key advantage of using human tissue is that it not only removes cross-species heterogeneity in tissue responses to injury and drug intervention, but it also retains native tissue architecture and cell-cell signalling pathways that are lost with the study of isolated cells.

K_Ca_3.1 ion channels are a promising target for IPF. These Ca^2+^-activated K^+^ channels regulate constitutive and TGFβ1-dependent pro-fibrotic activity in human lung myofibroblasts by promoting Ca^2+^ influx, SMAD2/3 phosphorylation and nuclear translocation, α-smooth muscle actin (αSMA) expression/stress fibre formation, and cell contraction^20–22^. K_Ca_3.1 channels promote bleomycin-dependent lung parenchymal fibrosis in sheep^23^, and airway fibrosis in a mouse model of asthma^24^, as well as tissue fibrosis in other organs^25,26^. A selective K_Ca_3.1 blocker (senicapoc [ICA-17043]) administered orally was very well tolerated over 12 months in a human phase III clinical trial for sickle cell disease^27,28^.

In light of the above, we hypothesised that healthy lung parenchyma obtained from lung resection specimens stimulated with TGFβ1 *ex-vivo*, would recapitulate proximal TGFβ1-dependent pro-fibrotic events evident in IPF lungs, and that these would be inhibited by the selective K_Ca_3.1 blocker senicapoc, but not dexamethasone. Part of this work has been presented previously in abstract form^29,30^.

## Results

### Assessment of tissue viability and response to *ex vivo* culture

**Figure 1:**
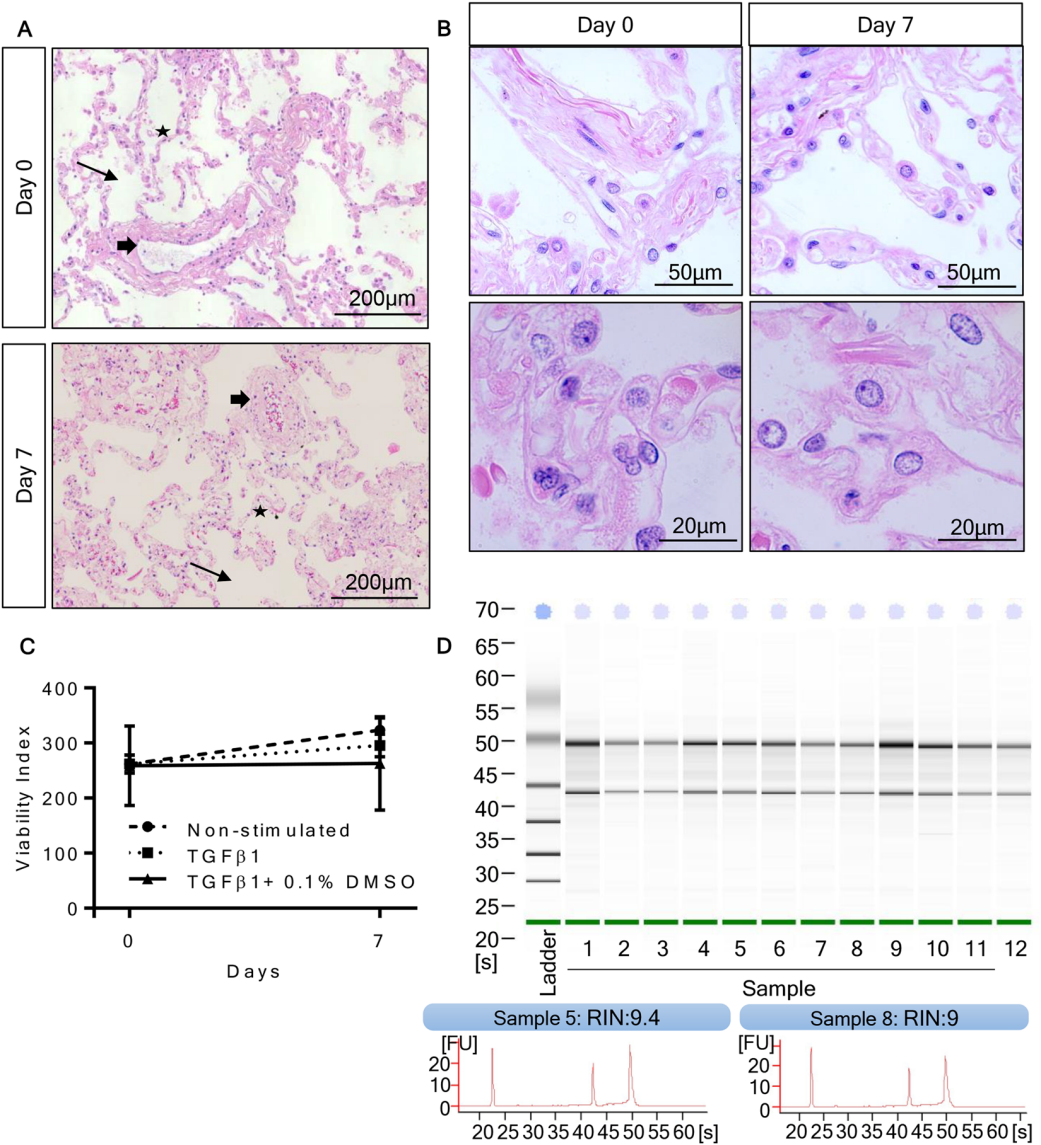
Tissue viability after 7 days of *ex vivo* culture. (**A**) Representative H&E staining of tissue embedded on day 0 or after culture for 7 days in serum free medium, from the same donor. These images highlight that alveoli spacing (thin arrow), single cell alveoli walls (star), and vessel structures (thick arrow) remain intact after 7 days of *ex vivo* culture, with no visible signs of tissue necrosis. (**B)** Magnified images demonstrate preserved nuclear morphology with no sign of nuclear fragmentation. (**C)** Viability of the tissue was assessed using the MTS assay at day 0 and day 7. Tissue metabolic activity was similar at day 0 and day 7 in control tissue, and in the presence of TGFβ1 (10 ng/ml) or TGFβ1 ± 0.1% DMSO (n = 19). (**D)** Representative bioanalysis of RNA samples demonstrating crisp, clear 2:1 ratio’s of ribosomal RNA bands at 28 S to 18 S, and RIN values of all samples was >8.

Lung tissue viability was assessed after 7 days of *ex vivo* culture by examining nuclear morphology, tissue necrosis, metabolic activity, and RNA quality, and compared to tissue at day 0. Nuclear morphology showed no signs of fragmentation and there were no visible signs of tissue necrosis (Fig. 1A and B). Alveolar structures were still identifiable. The MTS assay was used to determine the number of metabolically active cells present in tissue in media ± TGFβ1 (10 ng/ml) ± 0.1% DMSO at day 0 and day 7. Tissue metabolic activity was not significantly affected over the course of the 7 day culture, indicating that the number of viable cells was similar in all conditions (Fig. 1C). RNA isolated from days 0 and 7 was assessed for quality, and 100% of the samples (n = 10 paired donors) were suitable for gene expression arrays. The total RNA isolated from the explanted tissue samples had an RNA integrity number [RIN] of >8 (Fig. 1D).

We assessed differences in tissue fibroblast numbers and collagen staining between days 0 and 7 using immunohisotchemistry. Collagens type I and III, and αSMA were quantified as % area stained, and FSP+ fibroblast-like cells counted (fibroblasts were distinguished from macrophages by morphology). No significant differences in collagen deposition, αSMA expression or fibroblast-like cell numbers were found (Supplementary Figure 1A–E **)**. The presence of other cell types such as mast cells, macrophages were confirmed in both day 0 and day 7 cultures. Mast cell density assessed by immunohistochemistry for tryptase was 22.0 ± 4.7 cells/mm^2^ at day 0, and 24.8 ± 5.6 cells/mm^2^ at day 7, p = 0.65.

Gene expression in tissue was compared between day 0 and day 7 tissue using the Qiagen RT^2^ profiling gene array (n = 9 donors). Of 84 genes examined, only 9 were significantly differentially expressed (5 upregulated, 4 downregulated, Supplementary Table 1).

Therefore serum-free medium containing minimal essential nutrients is sufficient for the maintenance of viable, quiescent normal pulmonary parenchymal cells and architecture in *ex-vivo* lung tissue cultured for 7 days.

### Modelling lung fibrogenesis in 7 day *ex vivo* culture

**Figure 2:**
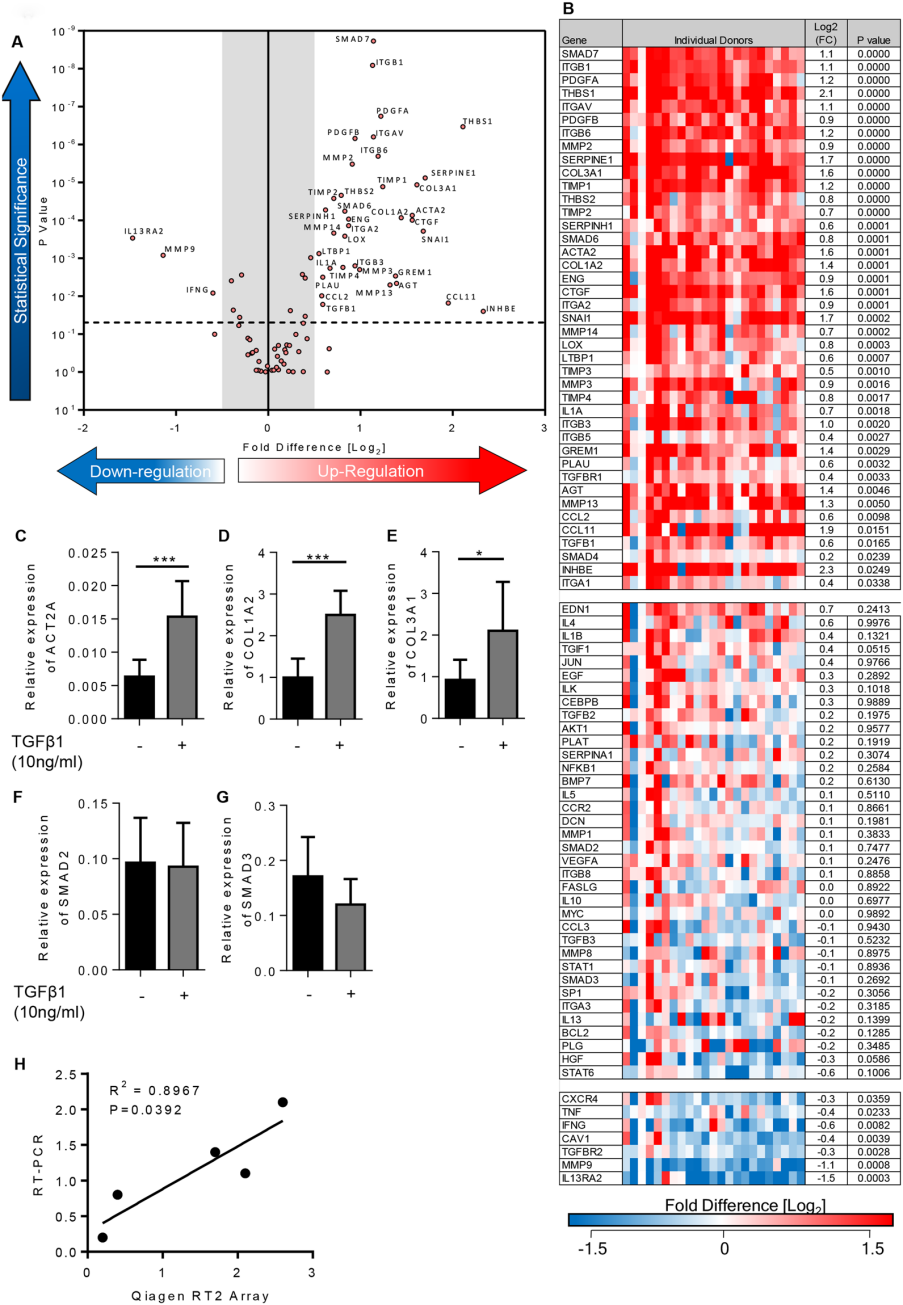
Differentially expressed genes in 7 day TGFβ1-stimulated lung tissue compared to control tissue. (**A**) Volcano plot identifying the statistically significant genes (p value) versus fold regulation (Log_2_(FC)) on the y and x axis respectively. Out of 84 genes, 41 were statistically significantly upregulated and 7 downregulated following a 5% false discovery analysis with John D. Storey adjusted p value < 0.05 and absolute value of log_2_(FC) ≥0.5, depicted by the dotted lines and grey shaded area (results are mean of 23 individual donors). (**B**) The log2 fold regulation of the 84 genes in individual donors is depicted in this heatmap, with mean log_2_(FC), and statistical significance indicated. Significance was calculated using a student’s t-test on the ∆∆CT for each gene. (**C**) Relative expression of ACT2A (αSMA mRNA) was significantly upregulated in TGFβ1-stimulated tissue in comparison to control tissue, P = 0.0010, n = 11. (**D**) COL1A2 (collagen type 1 mRNA) expression was significantly increased in TGFβ1-stimulated tissue in comparison to control, P = 0.0058, n = 11. (**E**) Relative expression of COL3A1 (collagen type III mRNA) was significantly upregulated in TGFβ1-stimulated tissue, P = 0.0296, n = 12. (**F**) SMAD2 mRNA expression was not significantly changed between control or TGFβ1-stimulated tissue after 7 days *ex vivo* culture P = 0.4961, n = 9. (**G**) Similarly, relative expression of SMAD3 mRNA expression was also not changed following 7 days TGFβ1-stimulation, P = 0.4656, n = 8. (**H**) When comparing the log_2_ fold regulation of the 5 genes there was a strong correlation with the results found using Qiagen RT^2^ profiling gene array and qRT-PCR using different primers, R^2^ = 0.8967, P = 0.0392. Results are presented as mean ± SEM, ***P < 0.01, *P < 0.05.

TGFβ1 has a pivotal and extensive role in the development of tissue fibrosis in IPF and other fibrotic disorders^8,9,31,32^. We therefore examined the tissue response to TGFβ1 (10 ng/ml) for 84 key genes implicated in human fibrotic processes, using the Qiagen RT^2^ PCR profiling array for human fibrosis. 41 pro-fibrotic genes were significantly upregulated in TGFβ1-stimulated tissue. All of these passed the false discovery rate (FDR) and 37 were upregulated with a log2-fold change (log_2_(FC)) of ≥0.5 (≥0.5 is considered biologically significant)(Fig. 2A and B). 7 genes were significantly down regulated (Fig. 2A and B); all passed the FDR and 3 were downregulated with a log_2_(FC) of ≤−0.5. Notably, ECM collagens type I and III were upregulated, and numerous ECM remodelling enzymes, such as MMP3 which is a biomarker of IPF severity^33^ and predictor of shortened survival time^34^. mRNAs for many relevant cell adhesion molecules were upregulated including ITGB6 and ITGAV, subunits of the αvβ6 integrin. αvβ6 is an important component of TGFβ1 activation pathways in experimental animal models of fibrosis^9,35^, is increased in IPF patients^36^, and correlates with a worse prognosis^36^. Key pro-fibrotic cytokines and chemokines were also upregulated including CCL11 (eotaxin); CCL11^−/−^ deficient mice have reduced bleomycin-induced pulmonary fibrosis and expression of pro-fibrotic cytokines such as TGFβ1 are diminished in the absence of CCL11^37^. Furthermore, several pro-fibrotic growth factors were upregulated, such as CTGF and PDGF, whose crosstalk with the TGFβ1 pathway are implicated in regulating fibrosis^38–40^. Interleukin-13 receptor alpha 2 (IL13RA2) was downregulated in this model of fibrogenesis which is notable as IL13RA2 is likely anti-fibrotic and plays a protective role in the lung by acting as a decoy receptor for IL-13^41^. Moreover, 16 of the 41 genes upregulated in this *ex vivo* model were upregulated in a previous study of IPF tissue, (Supplementary Table 2)^34^.

RT^2^ profiling gene array results were verified by individual qRT-PCRs^21,22^. The results were reproducible between methods and genes that were significantly increased using the RT^2^ profiling gene array (ACT2A, COL1A2, COL3A1), were increased to a similar extent using individual qRT-PCRs (Fig. 2C–E). Gene expression results were also consistent between techniques for genes that showed no significant change following 7 days of TGFβ1-sitmulation, SMAD2 and SMAD3(Fig. 2F,G **)**. Log_2_(FC) were strongly correlated, correlation coefficient R^2^ = 0.8967 (Fig. 2H **)**.

**Figure 3:**
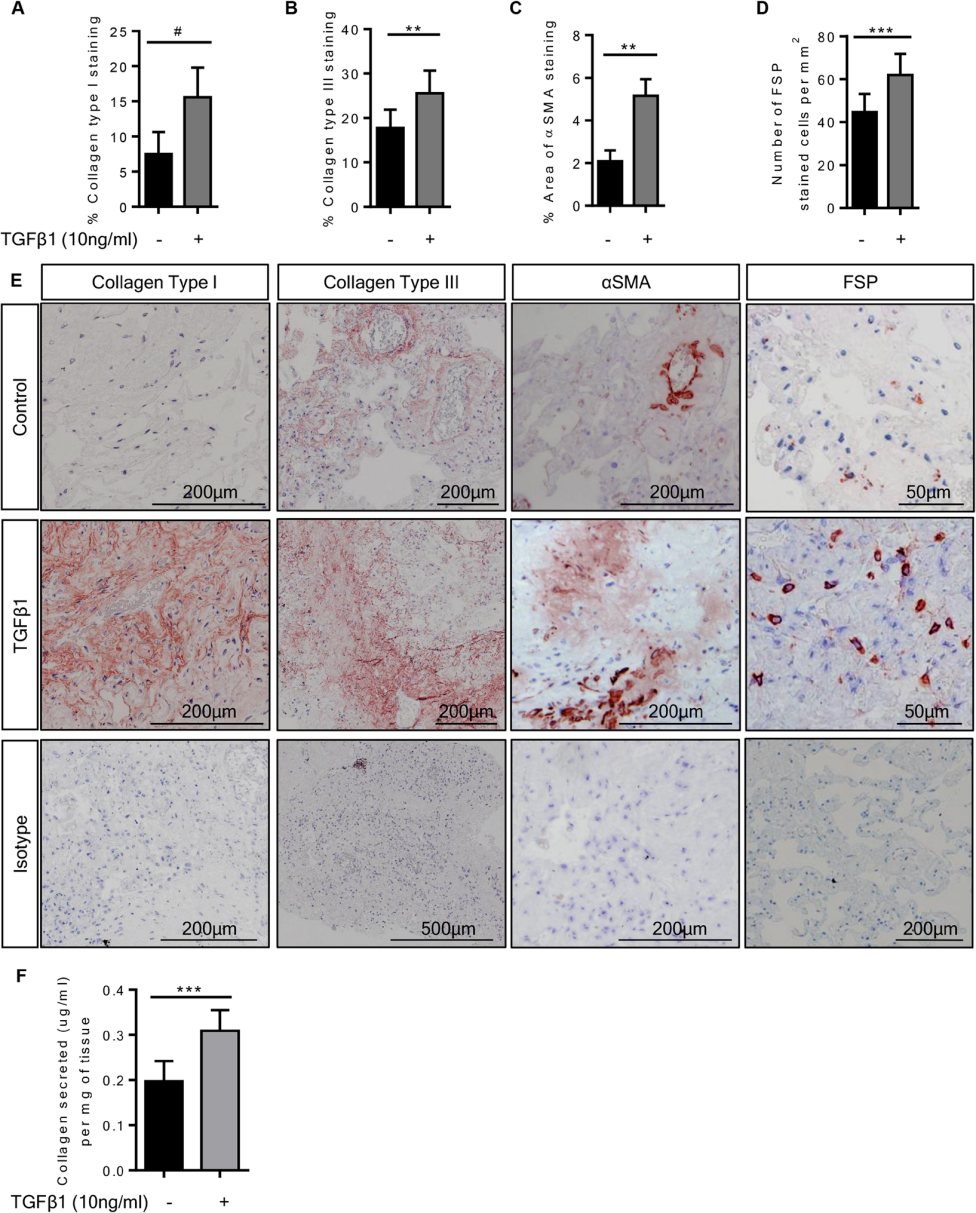
Immunohistochemical analysis of TGFβ1-stimulated tissue compared to matched control tissue. (**A**) The percentage collagen type I staining in TGFβ1 (10 ng/ml)-stimulated tissue was significantly greater than in control tissue following 7 days of *ex vivo* culture, P = 0.0002, n = 13. (**B)** Similarly, collagen type III staining was increased in TGFβ1-stimulated tissue, as measured by percentage area stained, P = 0.0017, n = 13. (**C**) αSMA staining was significantly higher in TGFβ1-stimulated tissue, P = 0.0012, n = 13. (**D)** The number of fibroblast-like cells assessed by counting cells stained with FSP antibody was increased following 7 days of TGFβ1-stimulation in comparison to control tissue, P = 0.0006, n = 13. (**E**) Representative immunohistochemical staining is depicted for each antibody for both control and TGFβ1-stimulated tissue following 7 days of *ex vivo* culture, including representative images of relevant isotype controls. (**F)** Supernatants following 4 days of *ex vivo* culture were analysed for collagens type I to V using the Sircol dye base method. The amount of collagen secreted (µg/ml) was assessed per total weight of tissue present in the well (mg). Collagen was significantly increased in supernatants from TGFβ1-stimulated tissue in comparison to non-stimulated tissue following 4 days of *ex vivo* culture, P < 0.0001, n = 15. Data are presented as median ± IQR, #P < 0.05, (Wilcoxon matched pairs signed rank test), or mean ± SEM **P < 0.01, ***P < 0.001 (paired t test).

Immunohistochemistry was used to examine the tissue protein expression of selected upregulated genes and other pro-fibrotic markers. When we treated the tissue with TGFβ1 (10 ng/ml) for 7 days and compared this with control day 7 tissue, we observed significant increases in the expression of the ECM molecules collagens type I and III, αSMA and the numbers of FSP+ fibroblast-like cells present in the tissue (Fig. 3A–E **)**. These findings are in keeping with other *in vitro* models of fibrosis^21,42,43^ and animal bleomycin models of fibrosis^44,45^.

Collagen secretion was also assessed in the supernatants collected after 4 days of *ex vivo* culture using the Sircol method, which utilises dye-binding for the analysis of newly synthesised ECM by quantifying acid and pepsin soluble collagens types I to V. After 4 days of *ex vivo* TGFβ1 exposure, collagen secretion into the supernatants was significantly increased which is in accordance with the ECM genes that were upregulated (Fig. 3F).

Thus, this *ex vivo* model of human lung fibrogenesis recapitulates many molecular features identified in the lung tissue of individuals with IPF.

### The selective K_Ca_3.1 blocker Senicapoc (ICA-17043) attenuates TGFβ1-dependent fibrogenesis in human lung parenchyma

**Figure 4:**
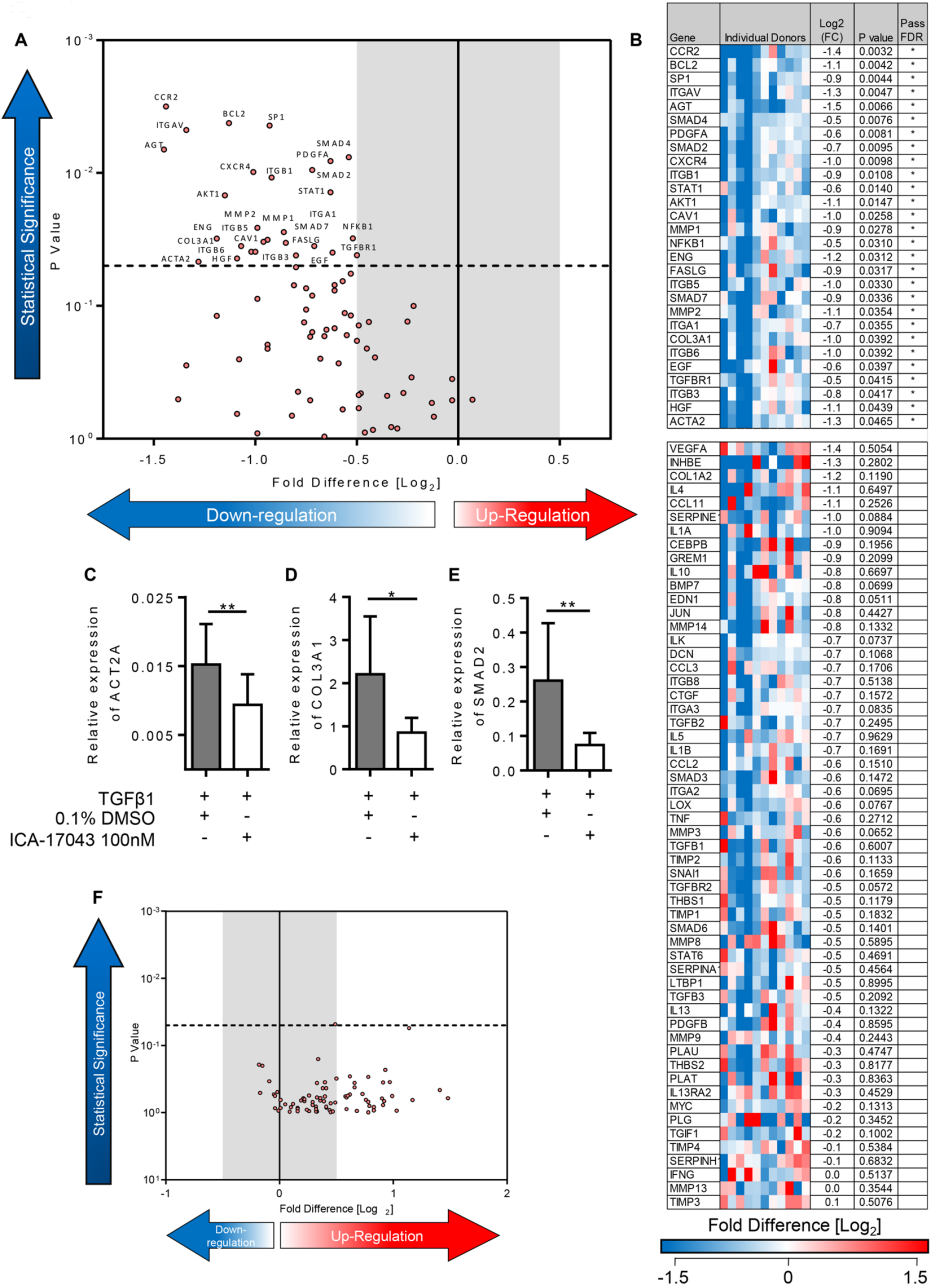
K_Ca_3.1 modulation of TGFβ1-induced fibrotic gene expression in an *ex vivo* model of fibrogenesis. (**A)** Volcano plot demonstrating that out of 84 genes, 28 were significantly downregulated following treatment with Senicapoc for 7 days in comparison to TGFβ1-stimulation alone. A 5% false discovery analysis with John D. Storey adjusted p value < 0.05 and absolute value of log_2_(FC) greater than 0.5 was used, depicted by the dotted lines and grey shaded area (results are mean of 11 individual donors). (**B)** The individual fold regulation of 84 genes is depicted in this heatmap, highlighting the magnitude of genes downregulated following treatment with senicapoc (100 nM) for 7 days. (**C**–**E**) Relative expression of ACT2A, COL3A1 and SMAD2 were significantly downregulated when treated with senicapoc, P = 0.0098, P = 0.0210, and P = 0.0078 respectively, consistent with RT^2^ profiling gene array results, n = 11. (**F**) No differences between TGFβ1 and TGFβ1+ 0.1% DMSO (vehicle control) were found using RT^2^ profiling gene arrays as displayed in the volcano plot identifying the gene expression of 84 fibrosis-associated genes. Results are presented as mean ± SEM, ***P < 0.01, *P < 0.05.

We predict that studying fibrogenesis in human tissue may prove effective for identifying drugs that have efficacy in IPF. K_Ca_3.1 channels are a promising therapeutic target. Consistent with its ability to suppress pro-fibrotic activity in myofibroblasts, and structural changes within animal models of fibrosis, the selective K_Ca_3.1 blocker senicapoc (100 nM [10x the Kd for channel block]) significantly attenuated the TGFβ1-dependent upregulation of pivotal genes implicated in the development of IPF (n = 11 donors)(Fig. 4A and B). Whereas 41 pro-fibrotic genes were upregulated by TGFβ1 compared to control, only 12 genes were upregulated by TGFβ1+ senicapoc compared to control (Supplementary Table 3).

When compared to TGFβ1+ 0.1% DMSO, 28 genes were significantly downregulated by senicapoc (Fig. 4A and B). These included ECM proteins (COL3A1) and myofibroblast markers (αSMA). Senicapoc also down regulated many cell adhesion molecules (ITGB6), pro-fibrotic growth factors (PDGFA), inflammatory cytokines and chemokine receptors (CCR2), matrix metallopeptidases (MMP2) and signal transduction and transcription factors (SMAD7, STAT1). These findings were supported by the use of selected RT-PCRs using custom primers for αSMA, collagen type III and SMAD2 (Fig. 4C–E). DMSO 0.1% did not have any effect when added to TGFβ1 (Fig. 4F).

**Figure 5:**
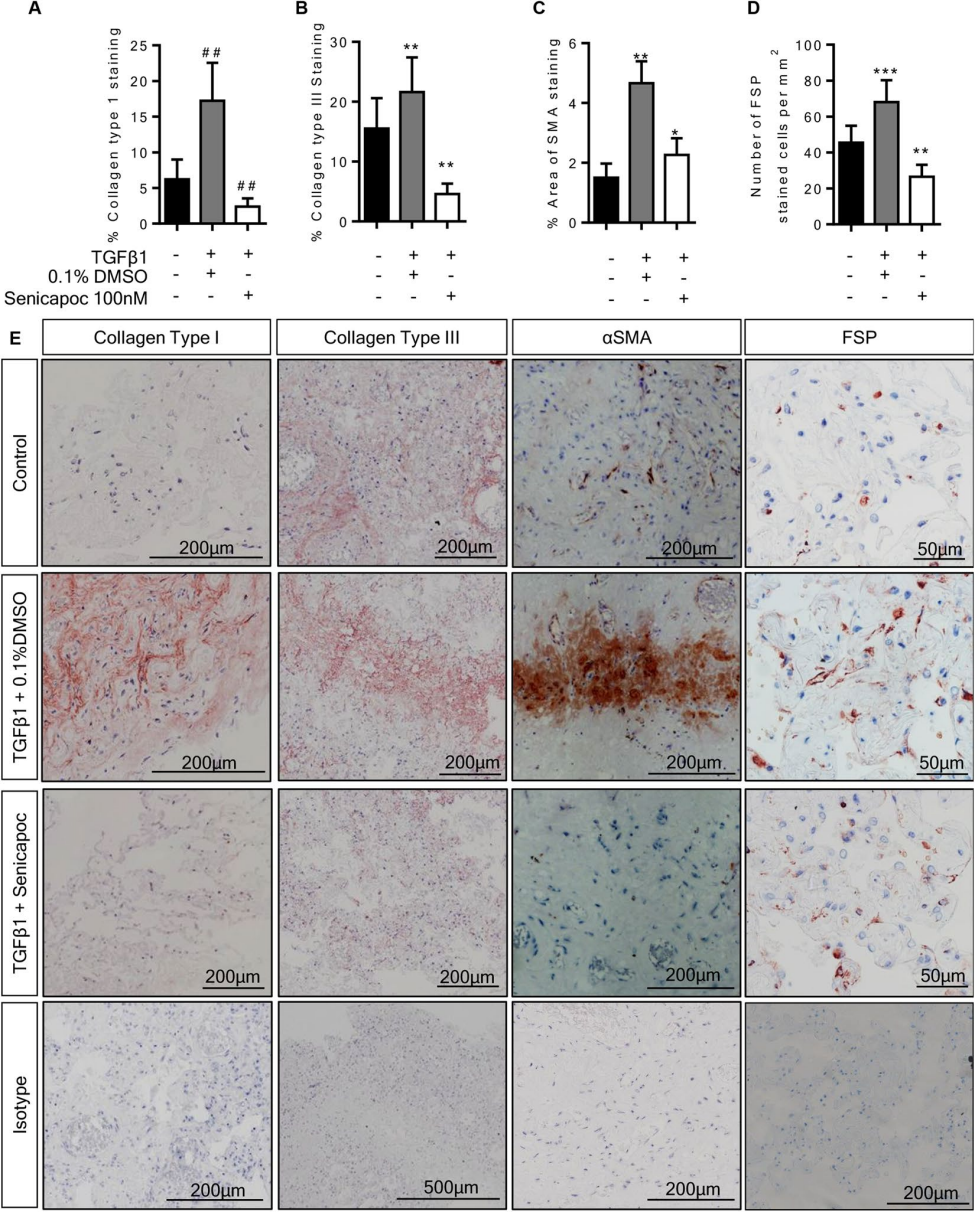
Senicapoc inhibits TGFβ1-induced pro-fibrotic structural responses in the 7 day *ex vivo* tissue model. (**A**) Analysis of production of collagen type I, collagen type III, αSMA, and fibroblast cell numbers following exposure to TGFβ1 with or without the K_Ca_3.1 inhibitor senicapoc (100 nM). 0.1% DMSO was used as vehicle control. Senicapoc significantly attenuated TGFβ1-dependent pro-fibrotic responses within the tissue over 7 days *ex vivo* culture, n = 10 individual donors. Data are presented as median ± IQR, ^##^P < 0.01 (Wilcoxon matched pairs signed rank test), or mean ± SEM **P < 0.01, ***P < 0.001 (paired t test). (**B**) Representative immunohistochemical images of 7 day *ex vivo* culture tissue under various conditions, un-stimulated control, TGFβ1-stimulated plus 0.1% DMSO and TGFβ1-stimulated treated with senicapoc (100 nM). Control and treated tissues were matched.

Immunohistochemical analysis supported the results from the profiling gene array, with reduced expression of collagens type I and III and αSMA, and a reduction in the number of FSP+ fibroblast-like cells within the tissue following 7 days of *ex vivo* culture with senicapoc compared to TGFβ1 alone (Fig. 5A–E). Staining was performed in paired sequential sections for FSP/αSMA, and collagen type 1/collagen type III, low power images of the same regions are depicted in Supplementary Figures 2 and 3.

### Dexamethasone does not inhibit TGFβ1-dependent fibrogenesis

**Figure 6:**
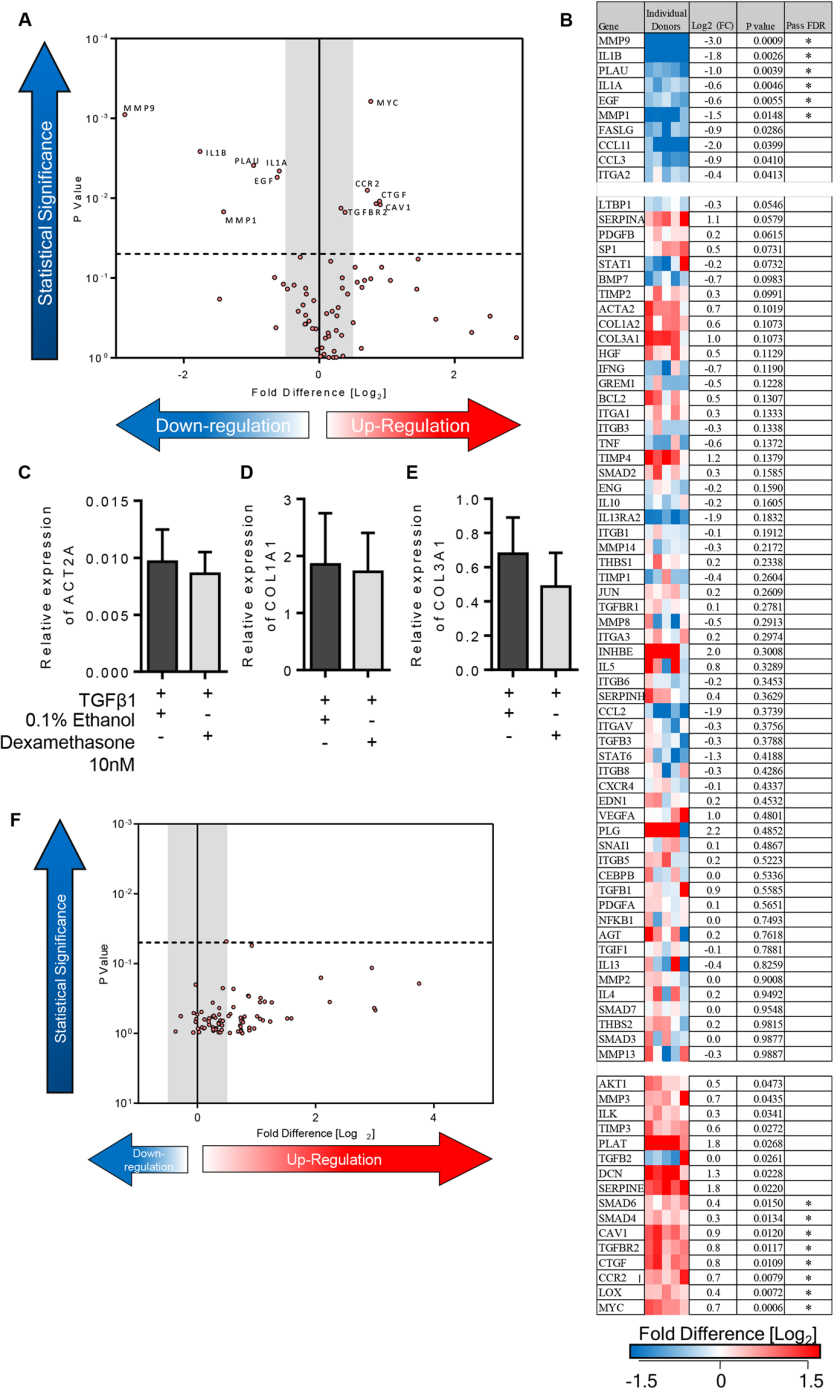
The effects dexamethasone on TGFβ1-induced fibrotic gene expression. (**A**) Volcano plot displaying the 11 differentially expressed genes following treatment with dexamethasone for 7 days in comparison to TGFβ1 alone, n = 5 donors. Treatment with dexamethasone resulted in the upregulation of 6 genes and downregulation of 8 genes passing 5% false discovery analysis with John D. Storey adjusted p value < 0.05 and absolute value of Log_2_(FC) greater than 0.5, depicted by the dotted lines and grey shaded area (results are mean of 5 individual donors). (**B**–**D**) Relative expression of ACT2A, COL1A1 and COL3A1 were unchanged when the TGFβ1-stimulated tissue was cultured with dexamethasone (10 nM) (**F)** Similarly, 0.1% ethanol (vehicle control for dexamethasone) did not affect TGFβ1-stimulated fibrogenesis highlighted in the volcano plot identifying the gene expression of 84 fibrotic genes.

Corticosteroids are widely believed to be ineffective in IPF, and in combination with azathioprine increase mortality in IPF^46^. In contrast to the results for senicapoc, the corticosteroid dexamethasone (10 nM) had no overall effect on TGFβ1-induced fibrogenesis (n = 5)(Fig. 6A and B), likely due to its known failure to inhibit TGFβ activation pathways^47^. However, as expected, dexamethasone did inhibit mRNA for pro-inflammatory molecules such as inflammatory cytokines (IL-1α, IL-1β), similar to results from previous studies^48,49^. In summary, out of 84 genes examined in the presence of TGFβ1, dexamethasone downregulated 6 genes which passed the FDR, and upregulated 8 pro-fibrotic genes at ≥0.5 log_2_(FC) which passed the FDR, including CTGF. These findings were supported by the use of selected RT-PCRs using custom primers for αSMA and collagens type I and III (Fig. 6C–E). Ethanol vehicle (0.1%) was without effect (Fig. 6F).

### Effect of pharmacological intervention on collagen secretion

**Figure 7:**
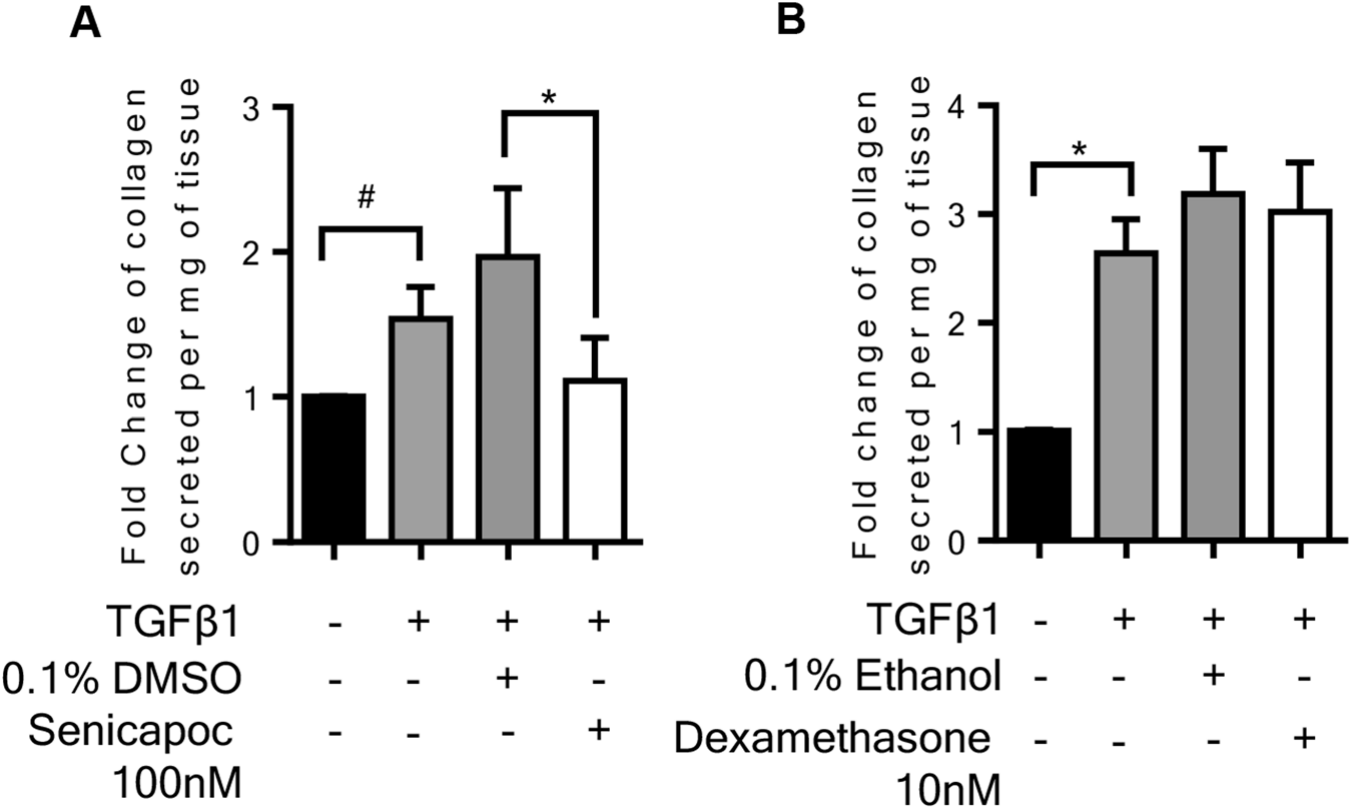
Pharmacological intervention in the fibrogenesis model has varying effects on collagen secretion after 4 days *ex vivo* culture. (**A**) K_Ca_3.1 blockade significantly attenuated collagen secretion into the supernatants following 4 days of *ex vivo* culture. The amount of collagen secretion was examined per mg of tissue present in the well, n = 5. (**B**) Dexamethasone (10 nM) did not inhibit TGFβ1-stimulated collagen secretion into the supernatants following 4 days of *ex vivo* culture n = 5. Data are presented as median ± IQR ^#^P < 0.05 (one sample t test), or mean ± SEM *P < 0.05, (1-way ANOVA, corrected by Dunns multiple comparison test).

Collagen secretion was assessed in the supernatants collected after 4 days of *ex vivo* culture with TGFβ1 ± pharmacological intervention. Senicapoc was very effective at reducing the TGFβ1-dependent release of soluble collagen into the culture supernatants, whereas dexamethasone was ineffective (Fig. 7A and B).

### Pharmacological intervention and key effector genes in TGFβ1-driven fibrogenesis

To further analyse the RT^2^ profiling data we determined the key genes driving the fibrotic response in the human tissue model. We used factor analysis to investigate whether there were distinct groups of genes (factors) regulating TGFβ1-induced fibrogenesis, using the TGFβ1-dependent gene expression results from 23 donors. Three factors were found that represented three sets of genes regulated by TGFβ1 (Table 1). These three factors were emphasised when examining the effects of pharmacological intervention. Senicapoc significantly inhibited 6 (out of 14) genes in factor 1 and 3 (out of 9) genes in factor 2 (Table 1). Furthermore, dexamethasone, which has no clinical efficacy in IPF, inhibited 1 (out of 14) genes in factor 1 but actually significantly upregulated 4 genes across factors 1 and 2. These results give some insight into how various pharmacological treatments mechanistically affect fibrotic processes in human lung tissue through inhibition of specific gene groups that can be determined by factor analysis. We hypothesise that a treatment which attenuates nearly all of the TGFβ1-dependent genes on each factor may be particularly effective for the treatment of IPF.

**Table 1:** Factor analysis of TGFβ1-dependent fibrogenesis.

Genes	Factor 1	Factor 2	Factor 3	Senicapoc	Dexamethasone
TGFBR1	0.977			**↓**	
SMAD4	0.968			**↓**	**↑**
SMAD7	0.913			**↓**	
TIMP2	0.876				
PLAU	0.876				**↓**
SNAI1	0.865				
TGFBR2	0.840				**↑**
ITGB1	0.813				
ITGA1	0.766			**↓**	
THBS1	0.758				
TIMP1	0.745				
ITGB3	0.724			**↓**	
THBS2	0.722				
CXCR4	0.712			**↓**	
LOX		0.748			**↑**
ACTA2		0.885		**↓**	
CAV1		0.944		**↓**	**↑**
COL1A2		0.979			
CTGF		0.915			
ENG		0.866		**↓**	
CCL2		0.848			
ITGB5		0.927			
CCL11		0.782			
TIMP3			0.874		
TIMP4			0.911		
SERPINE1			0.780		

## Discussion

TGFβ1 is a key growth factor driving tissue fibrosis in IPF^9,10,32^. We have demonstrated that *ex vivo* human lung parenchyma remains viable in tissue culture for at least 7 days, and in the presence of TGFβ1, upregulates many pro-fibrotic genes that are also upregulated in human IPF tissue^34,36^. This is accompanied by an increase in the number of tissue myofibroblasts and increased deposition of collagens type I and III demonstrated by immunohistochemistry. Importantly, these “fibrotic” TGFβ1-dependent changes were sensitive to pharmacological intervention with a selective blocker of the pro-fibrotic K_Ca_3.1 ion channel.

We believe this model has several advantages that will allow it to augment those currently used to study lung fibrosis. Firstly, by studying fibrotic events over 7 days, this reduces experimental time significantly over *in vivo* bleomycin studies. Secondly, the model avoids the relatively high financial costs and logistics of animal experimentation. In contrast, this model uses excess tissue removed at surgery, and is relatively cheap. Thirdly, the use of human tissue removes the issue of cross-species heterogeneity which is difficult to account for when interpreting animal experiments designed to study human disease processes. Lastly, in contrast to the study of isolated cells or bioengineered tissues, the use of native human tissue explants maintains the complex 3D cell-cell and cell-matrix interactions present in human lung.

While this human lung model undoubtedly has limitations when considering the prolonged course of human IPF, it nevertheless provides a means to study the consequences of TGFβ1 exposure in the 3D lung tissue microenvironment. Many of the molecules upregulated in our limited fibrosis gene array are upregulated in IPF tissue, including those for matrix proteins, MMPs, integrins (ITGAV, ITGB6), αSMA, and growth factors (CTGF, PDGFα). Human IPF progresses over time, and TGFβ1 signalling is believed to be the central driver^9,10,32^. Thus drugs which are able to inhibit the downstream consequences of TGFβ1-dependent signalling have the potential to protect the remaining healthy lung and thus halt disease progression, although reversal of established fibrosis is unlikely. This TGFβ1-dependent human lung model may therefore provide a reliable and rapid way of screening and identifying novel drugs for IPF treatment. Furthermore, the model provides the potential to study the downstream signalling networks activated by TGFβ1 in human parenchymal tissue, and how these are altered by drug exposure.

In human IPF tissue, FSP+ cells are predominantly fibroblasts^50^, although FSP is also expressed by tissue macrophages. For our analysis we only counted cells with a fibroblast-like morphology. It is also reported that murine endothelial cells and vascular smooth muscle cells express FSP^51^ but this was not evident in our human lung explants. The increase in the number of fibroblast-like cells expressing FSP in our study following TGFβ1 stimulation of lung tissue is therefore consistent with previous observations from IPF tissue, while the ability of senicapoc to inhibit this is consistent with its ability to inhibit the proliferation and differentiation of primary human lung myofibroblasts^20^.

In this study we have used immunohistochemistry and measurement of soluble collagen to validate selected gene expression changes identified by PCR array. We believe immunohistochemistry has advantages over other methods such as western blotting in that it is readily quantifiable using cell counts and image analysis, the analysis is highly reproducible, it allows analysis of tissue areas of interest, and subtle changes within tissue sub-compartments can be identified^52–55^. In contrast, western blotting of whole tissue extracts while semi-quantifiable, is open to marked confounding by the variable tissue components that might be present from sample to sample. For example, if one sample contains a large blood vessel, the αSMA actin will be greatly skewed. Using immunohistochemistry, such vessels can be readily excluded if not relevant to the analysis.

For this human lung model to be useful in drug development for IPF, it needs to be responsive to pharmacological intervention. Pirfenidone has been approved recently for use in IPF. It has modest efficacy, and attenuates the decline in lung function, although it’s mechanism of action is poorly understood. Nintedanib is also approved for IPF, with similar efficacy to pirfenidone. Currently these two drugs are the clinical benchmark with which to test potentially novel therapies in model systems. However the pathways and mechanisms involved in pirfenidone treatment are understudied and it is unclear how it is exerting its affects. This model therefore has the potential to improve understanding of the mechanism of action of pirfenidone in future studies, which in turn may lead to the development of improved medicines working through a similar mechanisms of action.

The K_Ca_3.1 ion channel promotes TGFβ1-dependent activation through the regulation of SMAD2/3 nuclear translocation and downstream transcription^20–22^. As a result, blocking or silencing K_Ca_3.1 inhibits the pro-fibrotic activity of parenchymal human lung myofibroblasts, and prevents tissue fibrosis in several animal models^25,26^, including bleomycin-dependent fibrosis in sheep^23^. In our current model, K_Ca_3.1 blockade using senicapoc, a drug very well tolerated in a phase 3 clinical trial of human sickle cell disease, was highly effective at inhibiting the pro-fibrotic effects of TGFβ1 both in terms of gene transcription, and tissue protein markers of fibrosis. Indeed, many genes known to be upregulated in IPF tissue were downregulated by senicapoc. Thus the current *in vitro* human evidence, the mouse and sheep *in vivo* data, and the favourable safety profile in human subjects suggest that clinical trials of K_Ca_3.1 blockade in IPF should be considered seriously.

In contrast to the effects of senicapoc, the corticosteroid dexamethasone was ineffective at inhibiting the effects of the pro-fibrotic features of TGFβ1 in this model, and upregulated some pro-fibrotic mediators. While corticosteroids alone have not been adequately assessed in randomised controlled clinical trials, there is evidence to suggest that they are ineffective in IPF^56,57^, and they increase mortality in combination with azathioprine^46^. There is therefore concordance between the inefficacy of corticosteroids in our model and their inefficacy in human IPF.

Alsafadi *et al*. recently published a model of human lung fibrogenesis similar to ours although they used precision cut lung slices on human lung inflated with agarose, and used a cocktail of TGFβ1, TNFα, PDGF-AB, and lysophosphatidic acid to activate pro-fibrotic pathways^58^. They demonstrated that their PCLS remained viable for at least 5 days assessed by tissue morphology and metabolic activity. Using qRT-PCRs for a few selected genes they demonstrated the upregulation of *FN1*, *SERPINE1*, *COL1A1*, *CTGF*, *MMP7*, and *ACTA2*, supported by western blotting and immunohistochemistry for the respective proteins (n = 4–6 donors), and the measurement of soluble collagen in the supernatants. Their results are therefore broadly in keeping with ours although the analysis was more limited in terms of genes of interest and donor numbers. We have shown that the pro-fibrotic response to TGFβ1 is consistent across a large number of donors although some donor-specific heterogeneity is inevitably present, and demonstrate that the model is sensitive to pharmacological intervention. Furthermore, Alsafadi *et al*. acknowledged a key limitation of their study was the lack of significant αSMA induction due to short culture times of PCLS. In our model 7 days culture was sufficient to see ECM remodelling and induce pathological αSMA changes. We believe that the major advantage of our model is the simplicity, with avoidance of inflation with agarose. Lung inflation with agarose requires access to intact lobes, but these are often not intact once the area affected by tumour has been removed for pathological assessment. In contrast, our model can be applied to distal lung parenchyma unsuitable for inflation.

A recent study used tissue derived from IPF patients to examine the activity of a PI3K/mammalian target of rapamycin (mTOR) inhibitor and showed that this inhibited Akt phosphorylation^59^. Studying IPF tissue is clearly useful, but relatively difficult to obtain for *ex vivo* studies as its collection relies on the availability of “end-stage” lungs removed for transplantation. In contrast, the use of healthy areas of lung from surgery, predominantly for carcinoma, is relatively straightforward, with over 250 surgical lung procedures in our hospital alone per annum.

In summary, we believe our *ex vivo* model of human lung fibrogenesis has the potential to simplify and accelerate the evaluation of potential therapeutic targets and associated therapies for human lung fibrosis, while at the same time providing insight into the mechanism of drug action. Thus, this model may represent an invaluable new tool to facilitate the analysis of TGFβ1-driven fibrogenic mechanisms and advance pre-clinical biomarker and drug discovery for IPF. Lastly, this study further strengthens the case for undertaking clinical trials of the K_Ca_3.1 blocker senicapoc in IPF.

## Methods

### Tissue collection

Human lung tissue samples (n = 40) were obtained from healthy areas of lung from patients undergoing lung resection for carcinoma at Glenfield Hospital, UK. All patients gave written informed consent and the study was approved by the National Research Ethics service (references 10/H0402/12 and 07/MRE08/42). All methods were performed in accordance with the relevant guidelines and regulations. Samples obtained were anonymised and coded before use. Patient demographics are shown in Table 2.

**Table 2:** Demographics of tissue donors.

Characteristic	n = 40
Sex	
Male	18 (45)
Female	22 (55)
Age (yrs)	
Mean (±SEM)	65 ± 1.83
Range	31–82
Smoking history	
Never	5 (12.5)
Ex <12 months pre-operatively	6 (15)
Ex >12 months pre-operatively	17 (42.5)
Current	7 (17.5)
Unknown	5 (12.5)
Number of Pack Years	
Mean (±SEM))	39.3 ± 3
Range	0–70
Lung Function	
FEV_1_	1.826 ± 0.1242
FEV_1_ (% predicted)	72.3 ± 4.29
FVC	2.98 ± 0.1396
FVC (% predicted)	96.87 ± 3.92
FEV_1_/FVC ratio (%)	61.16 ± 3.172
Diagnosis	
Non small cell	33 (82.5)
Carcinoid	5 (12.5)
Combined neuroendocrine carcinoma	1 (2.5)
Lung volume reduction surgery	1 (2.5)

Note: data presented as n (%) or mean ± SEM.

Abbreviations: FEV1, forced expiratory volume in 1 second; FVC, forced vital capacity.

### Tissue Explant Culture model

Healthy areas of tissue from lung resection surgery were cut into approximately 2 mm^3^ pieces and 4 pieces of tissue per well were placed into 6 well plates. Once cut, tissue was submerged in 3 ml of Dulbecco’s modified eagles medium (DMEM) supplemented with antibiotic/antimycotic agents and non-essential amino acids. Explanted tissue was cultured for 7 days in various conditions; serum-free medium alone, or serum-free medium plus: TGFβ1 (10 ng/ml), 0.1% DMSO, TGFβ1 (10 ng/ml) + 0.1% DMSO, TGFβ1 (10 ng/ml) + senicapoc (100 nM), TGFβ1 + 0.1% ethanol, TGFβ1 + dexamethasone (10 nM). Separate plates and tissue were used for RNA experiments and immunohistochemistry experiments. On day 4, supernatants were collected and culture media refreshed. On day 7 tissue was harvested.

### RNA Extraction

Tissue was placed into RNA later and stored at either +4 °C for 1 week or −20 °C for up to 1 month before isolation. Tissue was then placed in 300 µl of Qiagen buffer RLT with β***-***mercaptoethanol and homogenised using Precellys® hard tissue homogenizing tubes in the Precellys® 24 tissue homogenizer (Bertin Technologies, Montigny-le-Bretonneux, France) under a protocol adapted for lung tissue samples; 2 cycles at 6400 rpm for 30 seconds. Total RNA was purified using the automated QIAcube with RNeasy Fibrosis Mini kit (Qiagen, CA, USA) according to the manufacturer’s instructions. The RNA concentration and quality was measured using the Bioanalyzer 2100 system (Agilent, CA, USA). All samples, including day 0 and day 7 samples presented high-quality RNA with RNA integrity numbers of >8. RNA concentrations were then measured using the Nanodrop 2000 (Labtech International, East Sussex, UK).

### RT^2^ Profiling PCR Fibrosis Array

cDNA was pre-amplified using the RT^2^ first strand cDNA kit, according to manufacturer’s instructions (SabBioscience, Qiagen). A RT^2^ profiler human fibrosis PCR array (PAHS-120ZA) was performed for quantitative PCR in the Strategene MX3000P system according to the manufacturer’s instructions. Each PCR array plate examines 84 genes and has performance and quality controls for reverse transcription, genomic DNA contamination and PCR reproducibility. Any plate that did not pass was re-run; all the plates included in this paper passed. Ct was defined as 41 for the ΔCt calculation when the signal was below detectable limits. The average CT values of beta-2-microglobulin (β2M) and beta actin (β-actin) housekeeping genes were used as normalising controls. Results were calculated using the 2^−ΔΔCt^ method^60^. A minimum and maximum log 2 fold change of +5 to −5 was applied.

### Real time PCR analysis

Quantitative Real-time PCR (qRT-PCR) was used to measure mRNA expression levels of αSMA (ACT2A), collagen type I (COL1A1), collagen type III (COL3A1), SMAD2 and SMAD3, as a validation of the array data. All primers were validated (primer efficiency confirmed to be between 90–105%), sequenced and have previously been published^21,22,61^. PCR products were run on a 1.5% agarose gel to confirm product size and each product was sequenced to confirm specificity of the primers. Gene expression was quantified using Brilliant SYBR Green QRT-PCR 1-Step master mix (Strategene, the Netherlands) in the Strategene MX3000p system. All expression data were normalized to β-actin using Quantitect primer assay primers (Qiagen, Germany), HS_ACTB_1_SG and corrected using the reference dye ROX. For each experiment >50% of the samples used were also used for the RT^2^ profiler human fibrosis PCR array.

### GMA embedding and immunohistochemistry

Tissue was fixed in ice-cold acetone containing 2 mM phenylmethylsulfonyl fluoride and 20 mM iodoacetamide overnight at −20 °C and embedded in GMA, cut and stained as previously described^62^, two sections at least 10 μM apart were stained for each antibody. Haematoxylin and eosin (H&E) staining was used to determine tissue morphology. Primary antibodies were used against the following antigens: anti-alpha smooth muscle actin (αSMA), clone 1A4 (0.7 µg/ml;, Dako UK, Ely, United Kingdom); anti-fibroblast surface protein (FSP), clone 1B10 (4 µg/ml; Sigma-Aldrich, Dorset, UK); anti-collagen type I (550346, 15 μg/ml, Millipore, Watford, UK); anti-collagen type III, clone FH-7A (3.125 µg/ml; Sigma-Aldrich, Dorset, UK); and appropriate isotype controls mouse IgG1, and mouse IgG2a (Dako). The sections were incubated with appropriate secondary antibody polyclonal rabbit anti mouse F(ab)2 biotinylated (2.5 µg/ml, Dako), incubated with streptavidin-biotin peroxidise complex detection system (Dako, UK) for 2 h, treated with chromogen aminoethylcarbazole (AEC) giving a red reaction product, and counterstained with Mayer’s haematoxylin.

### Image analysis and quantitative morphometry

#### Measurement of αSMA immunostaining area

Immunoreactivity for αSMA was measured by a single observer at ×200 magnification using and was expressed as the percentage area of lung parenchymal tissue stained. To allow for biopsy variation, for each condition 2 separate blocks were immunostained and analysed. The mean measurement from 2 sections per block, at least 10 µM apart was used to determine the final value.

#### Measurement of extracellular matrix deposition

For quantitative assessment of matrix deposition in the lung parenchyma, a thresholding technique previously developed on the basis of hue, saturation and intensity (HSI) was used as described^63^. Areas of immunostained collagen type I and type III were assessed by a single observer at ×200 magnification, in 2 sections at least 10 µM apart and expressed as percentage area of lung parenchymal tissue stained.

#### Determination of fibroblast numbers

Nucleated immunostained cells for FSP-1 (fibroblast-like cells and myofibroblast-like cells) were counted in the explant tissue at ×400 magnification and expressed per mm^2^ of lung parenchymal tissue. Macrophages stained with this antibody were excluded from cell counts and analysis. The mean cell count of 2 sections at least 10 µm apart was used to determine the final count. Counts were enumerated by 2 blinded observers with excellent agreement, reliability and repeatability (Cronbach’s Alpha intra-class correlation coefficient 0.906).

### Collagen Secretion

Soluble collagen in the supernatants after 4 days of tissue culture was quantified using the Sircol collagen assay (Biocolor, County Antrim, UK) according to manufacturer’s instructions.

### MTS Assay

Human lung tissue viability was assessed by the MTS (3-(4,5-dimethylthiazol-2-yl)-5-(3-carboxymethoxyphenyl)-2-(4-sulfophenyl)-2H-tetrazolium) assay (Promega, Wisconsin, USA), as per manufacturer’s instructions. At day 0, and day 7, tissue was removed from culture, placed into a 96 well plate along with 150 µl of serum free media and 30 µl MTS reagent. Plates were incubated for 2 hours at 37 °C and then each piece of tissue was removed from the well and weighed. The plate was then placed in the Enspire Multimode plate reader (PerkinElmer, Massachusetts, USA) at 490 nM for 0.1 second. The viability of the tissue was assessed using the equation below to determine the Viability Index number (equation 1). Experiments were performed in duplicate.

1 $${\rm{Viability}}\,{\rm{Index}}=\frac{{\rm{Absorbance}}\,(\mathrm{490}\,\mathrm{nM})}{{\rm{Tissue}}\,{\rm{weight}}\,({\rm{g}})}$$

### Statistics

The RT^2^ profiling gene array has in-built reproducibility controls and therefore only one plate per donor was run. Other experiments from an individual donor were performed in duplicate or triplicate and a mean value was derived for each condition. Data distribution across donors was tested for normality using the Kolmogorov-Smirnov test. For parametric data, the one-way ANOVA or repeated measures ANOVA for across group comparisons was used followed by the appropriate multiple comparison post hoc test; A paired t test was used for groups of only two conditions on the same donor. For non-parametric data, the Friedman test was used for across group comparison of paired data followed by the appropriate multiple comparison post hoc test; otherwise a Wilcoxon rank sum test was used. Graph Pad Prism (version 6, GraphPad Software, San Diego, CA, USA) was used for these analyses. A value of P < 0.05 was taken to assume statistical significance and data are represented as mean (±SEM) or median (±IQR).

False discovery analysis was performed on all RT^2^ PCR profiling array data to overcome the problems of multiple testing. This was undertaken using John D. Storey adjusted p-value < 0.05 and q-value of 0.05 (5% FDR) derived from the full list of p-values.

### Factor Analysis

Factor analysis was carried out in SPSS on the fold change data of the TGFβ1 derived expression. The Factor analysis was carried out to determine if patterns of expression could be found within the correlated gene expression and thus see if different groups of genes behaved differently within tissue when TGFβ1 was applied. Due to the large number of genes recorded compared to the number of donor, the principle component analysis method was used to determine the number of factors and their factor loading within the correlated expression. The number of factors was chosen based on the Kaiser criterion of factors having eigen values of greater than one and then keeping the factors that had a least one factor loading greater than 0.7 as factor loading below this are difficult to interpret.

## Electronic supplementary material

Supplementary material
