# Supplementary material for: A model of human lung fibrogenesis for the assessment of anti-fibrotic strategies in idiopathic pulmonary fibrosis
Source: Sci Rep. 2018 Jan 10;8:342. doi: 10.1038/s41598-017-18555-9 (PMC5762721; doi:10.1038/s41598-017-18555-9)
Supplement: Supplementary file 1 — Supplementary material [file 41598_2017_18555_MOESM1_ESM.pdf]

*Supplementary material for*

**A model of human lung fibrogenesis for the assessment of anti-fibrotic strategies in idiopathic pulmonary fibrosis**

Katy M Roach[1], Amanda Sutcliffe[1], Laura Matthews[1], Gill Elliott[1], Chris Newby [1], Yassine Amrani [1], Peter Bradding[1]

[1] Institute for Lung Health, Respiratory Medicine, Department of Infection, Immunity and Inflammation, University of Leicester, UK.

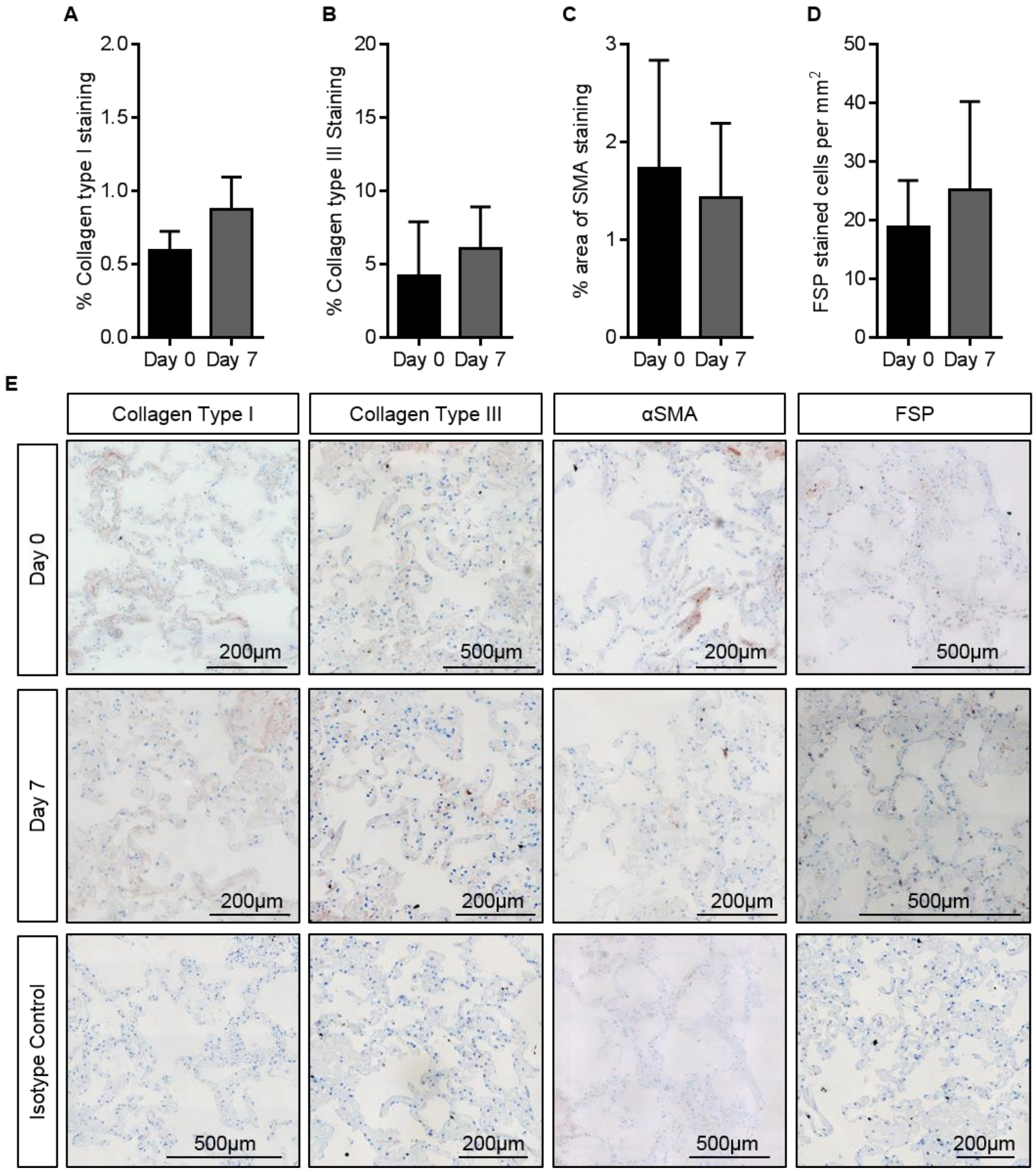


**Supplementary figure 1 – Histological differences of cellular staining in tissue following 7 days of *ex vivo* culture in comparison to day 0.**

**A)** Collagen type I staining was not significantly changed after 7 days in *ex vivo* culture, P=0.25 n=3. **B)** Similarly no differences in collagen type III staining was observed, P=0.75 n=3. **C)** The percentage area of αSMA staining was not affected following 7 days of *ex vivo* culture, P=0.75 n=3. **D)** No significant differences in the number of fibroblasts stained with fibroblast surface protein (FSP) at day 0 and day 7 were observed, P=0.5429 n=3. **E)** Representative images of collagen type I, collagen type III, αSMA, and FSP staining in day 0 and day 7 tissue. No significant differences were observed and relevant isotype controls were negative. Results are presented as mean± SEM.

**Supplementary figure 2 – Histological sequential FSP and αSMA staining in *ex vivo* cultured tissue following 7 days of with TGFβ1 and Senicapoc 100nM.**

Sequential staining of FSP and αSMA following 7 days ex vivo culture with TGFβ1 stimulated tissue and TGFβ1 + Senicapoc.

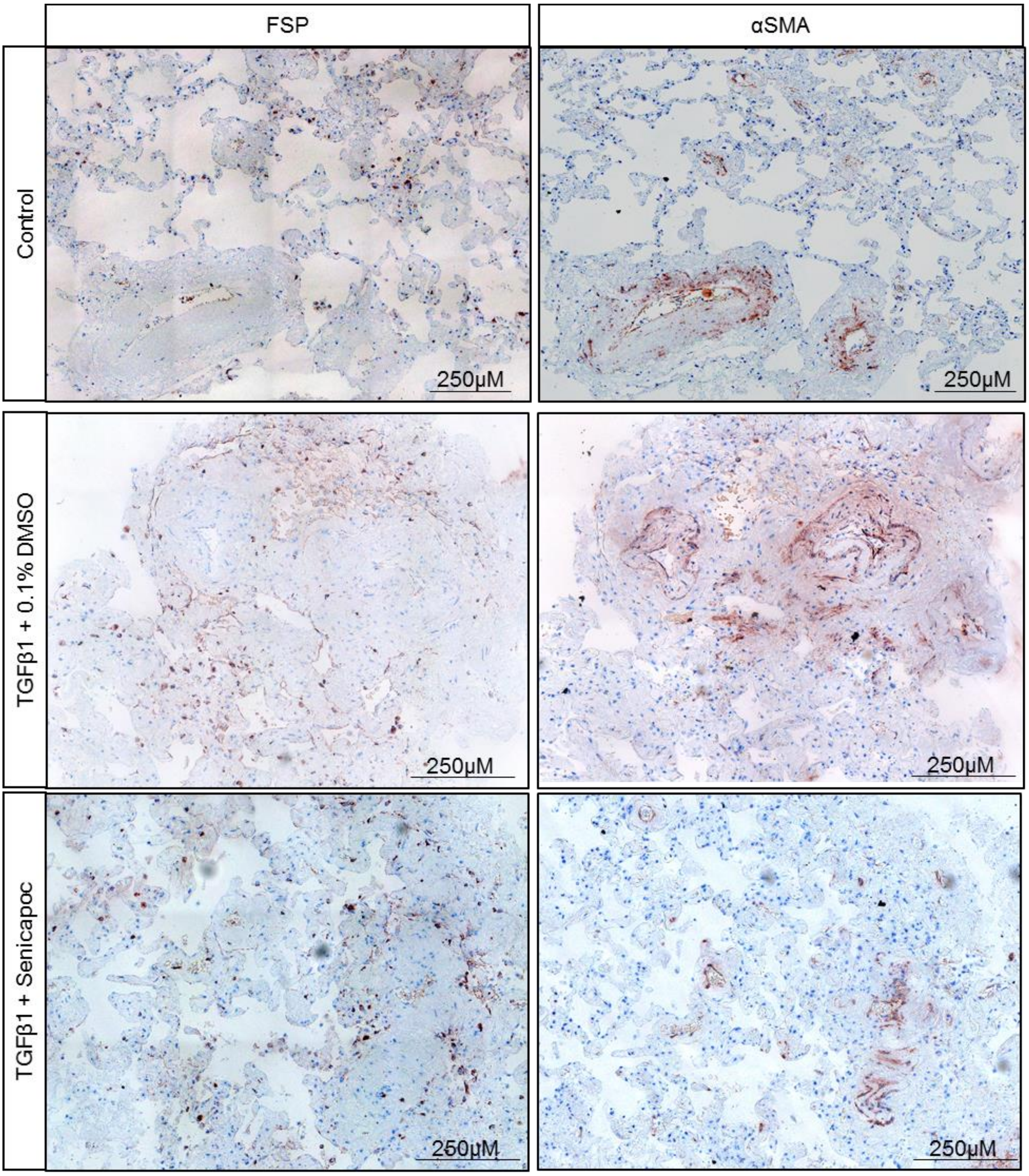

**Supplementary figure 3 – Histological sequential collagen type I and collagen type III staining in *ex vivo* cultured tissue following 7 days of with TGFβ1 and Senicapoc 100nM.**

Sequential staining of collagen type I and III following 7 days ex vivo culture with TGFβ1 stimulated tissue and TGFβ1 + Senicapoc.

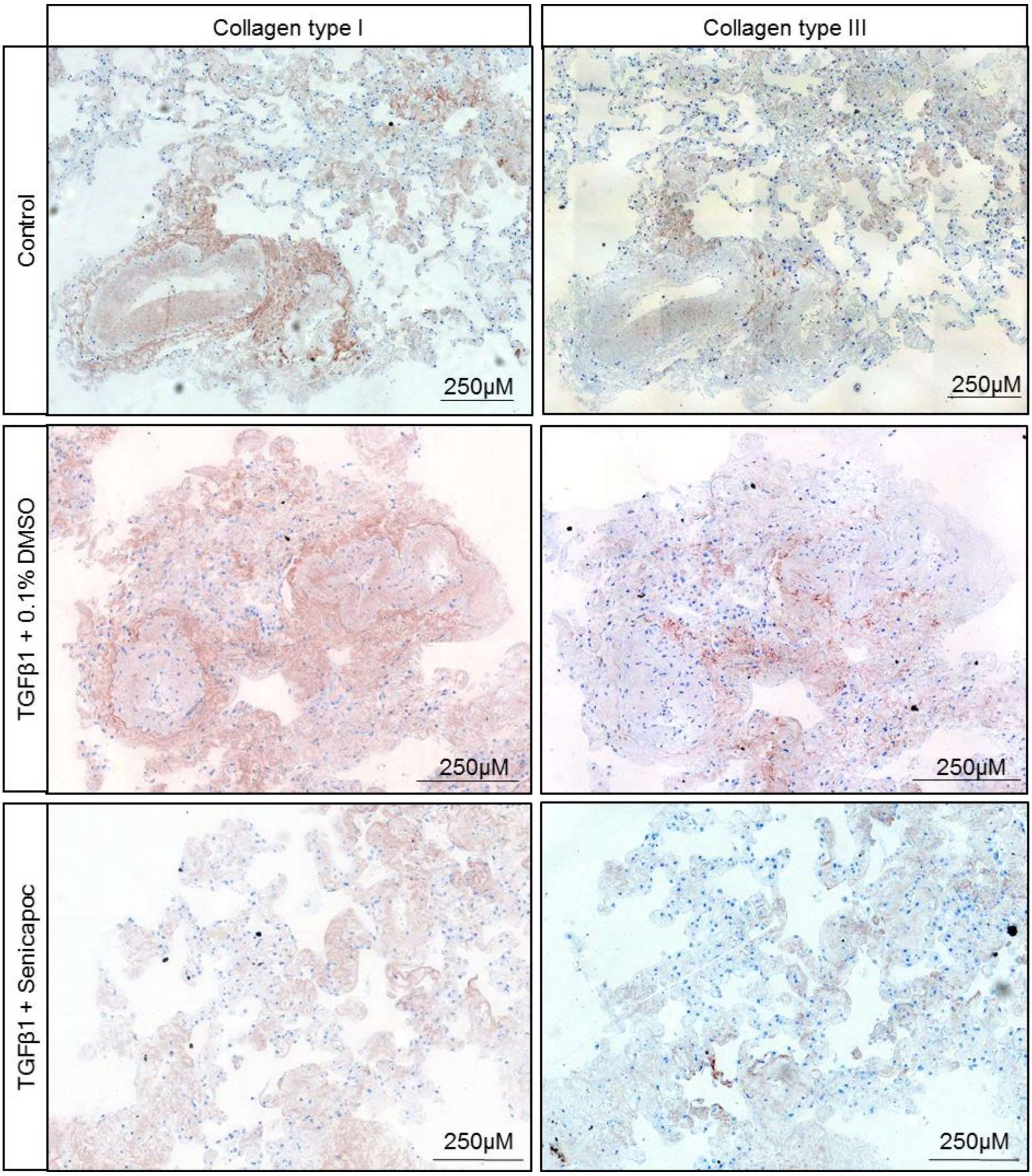

**Supplementary table 1 – Differentially expressed genes in day 7 vs. day 0 lung tissue**

| **Upregulated genes in day 7 explanted tissue compared to day 0 tissue** | | | | |
|---|---|---|---|---|
| **Gene Symbol** | **Gene Name** | **$Log_2$ FC** | **P-Value** | **Pass FDR** |
| MMP1 | Matrix metallopeptidase 1 | 4.0 | 0.0100 | * |
| TIMP1 | TIMP metallopeptidase inhibitor 1 | 3.0 | 0.0011 | * |
| THBS2 | Thrombospondin 2 | 2.9 | 0.0005 | * |
| LOX | Lysyl oxidase | 2.8 | 0.0022 | * |
| MMP9 | Matrix metallopeptidase 9 | 2.7 | 0.0374 | |
| MMP14 | Matrix metallopeptidase 14 | 2.7 | 0.0000 | * |

| **Downregulated genes in day 7 explanted tissue compared to day 0 tissue** | | | | |
|---|---|---|---|---|
| **Gene Symbol** | **Gene Name** | **$Log_2$ FC** | **P-Value** | **Pass FDR** |
| IL13RA2 | Interleukin 13 receptor, alpha 2 | 1.9744 | 0.0083 | * |
| CCL2 | Chemokine (C-C motif) ligand 2 | -1.414 | 0.0059 | * |
| CCL3 | Chemokine (C-C motif) ligand 3 | -1.559 | 0.0082 | * |
| IL5 | Interleukin 5 | -1.964 | 0.0259 | |
| SMAD6 | SMAD family member 6 | -2.14 | 0.0295 | |
| CCL11 | Chemokine (C-C motif) ligand 11 | -3.831 | 0.0066 | * |
| CTGF | Connective tissue growth factor | -4.211 | 0.0320 | |

Genes with a fold change ≥1.5, and John D. Storey adjusted p-value <0.05 in day 0 tissue compared to day 7 (n=9) are listed below. Using q-value of 0.05 (5% FDR).

**Supplementary table 2 – Gene expression results from the 7 day ex vivo human lung fibrogenesis model were comparable to that previously published in IPF tissue.**

| Gene Symbol | Gene Name | Fibrotic Model Tissue $Log_2$(FC) | IPF Tissue *(DePianto et al. 2015)* $Log_2$(FC) |
|---|---|---|---|
| THBS1 | Thrombospondin 1 | 2.1 | 0.9 |
| CCL11 | Chemokine (C-C motif) ligand 11 | 1.9 | 4.1 |
| COL3A1 | Collagen, type III, alpha 1 | 1.6 | 1.5 |
| COL1A2 | Collagen, type I, alpha 2 | 1.4 | 2.1 |
| GREM1 | Gremlin 1 | 1.4 | 4.2 |
| MMP13 | Matrix metallopeptidase 13 | 1.3 | 4.1 |
| TIMP1 | TIMP metallopeptidase inhibitor 1 | 1.2 | 0.9 |
| SMAD7 | SMAD family member 7 | 1.1 | -0.7 |
| ITGB3 | Integrin, beta 3 | 1.0 | 1.7 |
| MMP3 | Matrix metallopeptidase 3 | 0.9 | 2.3 |
| ENG | Endoglin | 0.9 | -0.6 |
| ITGA2 | Integrin, alpha 2 | 0.9 | 1.2 |
| SMAD6 | SMAD family member 6 | 0.8 | -1.6 |
| TIMP4 | TIMP metallopeptidase inhibitor 4 | 0.8 | -2.7 |
| THBS2 | Thrombospondin 2 | 0.8 | 2.0 |
| TIMP2 | TIMP metallopeptidase inhibitor 2 | 0.7 | 0.6 |
| PLAU | Plasminogen activator, urokinase | 0.6 | 1.7 |
| TGFB1 | Transforming growth factor, beta 1 | 0.6 | 0.8 |
| CCL2 | Chemokine (C-C motif) ligand 2 | 0.6 | 3.1 |
| LTBP1 | Latent transforming growth factor beta binding protein 1 | 0.6 | 1.2 |
| TIMP3 | TIMP metallopeptidase inhibitor 3 | 0.5 | -1.7 |
| TGFBR2 | Transforming growth factor, beta receptor II | -0.3 | -0.8 |
| CAV1 | Caveolin 1, caveolae protein, 22kDa | -0.4 | -1.2 |
| IL13RA2 | Interleukin 13 receptor, alpha 2 | -1.5 | 5.5 |

**Supplementary table 3 – Differentially expressed genes in TGFβ1 + Senicapoc stimulated tissue in comparison to control tissue.**

| Upregulated expressed genes in TGFβ1 + Senicapoc treated tissue vs control tissue | | | | |
|---|---|---|---|---|
| **Gene Symbol** | **Gene Name** | **$Log_2$ FC** | **P-Value** | **Pass FDR** |
| THBS1 | Thrombospondin 1 | 2.3 | 7.70E-04 | * |
| SNAI1 | Snail homolog 1 (Drosophila) | 2.1 | 4.20E-03 | * |
| SERPINE1 | Serpin peptidase inhibitor, clade E | 2.1 | 3.00E-03 | * |
| CTGF | Connective tissue growth factor | 2.0 | 1.54E-02 | * |
| PDGFA | Platelet-derived growth factor alpha polypeptide | 1.3 | 4.57E-03 | * |
| TIMP1 | TIMP metallopeptidase inhibitor 1 | 1.3 | 1.73E-03 | * |
| PDGFB | Platelet-derived growth factor beta polypeptide | 1.1 | 3.81E-02 | |
| ITGB1 | Integrin, beta 1 | 1.0 | 1.18E-02 | * |
| SMAD7 | SMAD family member 7 | 1.0 | 1.56E-02 | * |
| LOX | Lysyl oxidase | 0.9 | 2.05E-02 | * |
| VEGFA | Vascular endothelial growth factor A | 0.7 | 4.48E-02 | |
| IL1B | Interleukin 1, beta | 0.6 | 2.53E-02 | * |

| Downregulated expressed genes in TGFβ1 + Senicapoc treated tissue vs control tissue | | | | |
|---|---|---|---|---|
| **Gene Symbol** | **Gene Name** | **Log2 FC** | **P-Value** | **Pass FDR** |
| IL13RA2 | Interleukin 13 receptor, alpha 2 | -0.9 | 3.06E-03 | * |
| MMP9 | Matrix metallopeptidase 9 | -1.3 | 6.32E-03 | * |

## References


(1) Livak K, Schmittgen T. Analysis of relative gene expression data using real-time quantitative PCR and the 2(T)(-Delta Delta C) method. *Methods* 2001;25:402-408.

(2) Bradding P, Feather IH, Howarth PH, Mueller R, Roberts JA, Britten K, Bews JPA, Hunt TC, Okayama Y, Heusser CH, Bullock GR, Church MK, Holgate ST. Interleukin-4 is Localized to and Released by Human Mast-Cells. *J Exp Med* 1992;176:1381-1386.

(3) Siddiqui S, Mistry V, Doe C, Doe C, Roach KM, Morgan A, Wardlaw A, Pavord I, Bradding P, Brightling C. Airway hyperresponsiveness is dissociated from airway wall structural remodeling. *J All Clin Immunol* 2008;122:335-341e3.

(4) SPSS, IBM Corp. Released 2013. IBM SPSS Statistics for Windows, Version 22.0. Armonk, NY, America.
